# Characterization and application of the nanocompiste packaging films containing clay and TiO_2_ on preservation of tomato fruit under cold storage

**DOI:** 10.1186/s12870-024-05215-0

**Published:** 2024-06-10

**Authors:** Hojatollah Bodaghi

**Affiliations:** Department of Horticulture Science and Plant Protection, College of Agriculture, Shahrood University of Technology, Shahrood, Iran

**Keywords:** Nanocomposite packaging film, Clay, TiO_2_, Tomato, Storage life

## Abstract

**Background:**

Tomato (*Lycopersicon esculentum*), a valuable economic crop worldwide, often goes to waste due to improper packaging and handling. In the present study, three types of low-density polyethylene nanocomposite films containing 3% clay (Closite 20A), 3% TiO_2_ nanoparticles, and their combination were synthesized using melt blending method, and evaluated on the quality parameters of tomato fruit during 42 days of storage at 4 °C.

**Results:**

Transmission electron microscopy confirmed the degree of dispersion and exfoliation of the nanoparticles. The TiO_2_/clay-nanocomposite films exhibited notable enhancements in Young's modulus and tensile strength compared to conventional films. The addition of clay and TiO_2_ nanoparticles resulted in reduced permeability to CO_2_, O_2_, and water vapor. Fruits packed with clay/TiO_2_ nanocomposite films showed decreased ethylene production, mitigated weight loss, and maintained pH, titratable acidity, total soluble solids, and firmness. Furthermore, clay/TiO_2_ nanocomposite films enhanced membrane stability, decreased membrane lipid peroxidation, and enhanced catalase and ascorbate peroxidase enzyme activity in fruits.

**Conclusions:**

The relatively good exfoliation of clay nanoparticles and the proper dispersion of TiO_2_ nanoparticles, which were confirmed by TEM, led to an increase in mechanical and physical properties in the Clay/TiO_2_ nanocomposite. This film displayed more potential in maintaining the quality properties of tomato fruit during cold storage. Therefore, this film can be considered a practical solution for minimizing pathogen risks and contamination, and enhancing the overall quality of tomato fruit.

## Background

Tomato (*Lycopersicon esculentum* Mill.) is a globally cultivated fruit-bearing vegetable. It is widely utilized in both its fresh and processed forms, including canned products, sauces, tomato juice, ketchup, and soups. The susceptibility of tomatoes to spoilage is linked to their high water content, constituting approximately 90% of their fresh weight. Beyond their nutritional value, tomatoes harbor bioactive antioxidants beneficial for human health, notably lycopene. This compound, found in daily diets, holds the highest antioxidant activity among such components [1, 2].

Tomatoes are classified as climacteric fruits, where the ripening phase is characterized by an increase in the rate of respiration and the production of ethylene. This biological progression expedites the deterioration in fruit quality due to both physical and chemical changes, encompassing alterations in color and texture. Tissue firmness stands as a reliable quality indicator during the mature phase of tomato development, significantly influencing its market desirability. Generally, as tomatoes are stored, their firmness diminishes. The softening of the fruits is attributed to the degradation of cell wall carbohydrates and an escalation in soluble pectin content, inducing a weakening of cell walls and a decrease in adhesive forces between cells [3].

Numerous techniques have been introduced to counteract the detrimental influence of ethylene on perishable products. Photocatalytic oxidation has surfaced as a proficient means to eliminate ethylene from the ambient atmosphere surrounding fruits and ethylene-sensitive vegetables within storage facilities [4]. Recently, the application of photocatalytic oxidation for ethylene removal from tomatoes has been elucidated. This involves utilizing a continuous-flow reactor equipped with TiO_2_ as a semiconductor catalyst, in conjunction with ultraviolet light exposure [5]. The implementation of this process resulted in a decline of ethylene gas emission during the ripening phase, decreasing from 0.3182 μmolkg^−1^ h^−1^ to negligible levels when the reactor was subjected to ultraviolet light with an intensity of 5.18 Wm^−2^. Concurrently, the respiration rate of the fruit also demonstrated a reduction.

Controlled atmosphere (CA) and modified atmosphere packaging (MAP), coupled with reduced storage temperature, are suitable strategies to reduce respiratory intensity and ethylene levels, thereby slowing down the ripening process and extending the shelf life of products [6]. In the modified atmosphere packaging, over time, a composite of internal atmospheric gases is formed leading elevated CO_2_ levels and diminished O_2_ concentration, whereas in a CA, an appropriate gas composition of the mentioned gases is established and maintained at predetermined levels [7]. The beneficial effects of MAP for tomatoes were reported using perforated polypropylene films with a gas composition of CO_2_ by 3 kPa and O_2_ concentration below 12 kPa, along with a relative humidity of 90%, On the contrary, non-perforated films led to anaerobic conditions, resulting in a reduction in physicochemical properties of the product and loss of edibility quality [8]. In a study, greenhouse-packaged tomatoes were evaluated under modified atmosphere conditions (88% N_2_ + 8% CO_2_ + 4% O_2_) using two types of packaging films: low-density polyethylene (LDPE) and polyethylene containing nano-silicon, at a temperature of 5 °C for 30 days. The results of this research confirmed the extension of storage life and maintenance of fruit quality attributes until the end of the storage time in nano-silicon PE films compared to neat PE films [9].

Achieving success and satisfactory results in reducing physical and chemical changes, and spoilage caused by post-harvest pathogenic activities, selecting appropriate packaging is essential. However, weaknesses in factors like strength, tensile strength, thermal stability, gas permeability, solvent resistance, and antimicrobial properties of polyolefin plastic packaging films can limit their efficiency in the food packaging industry [10].

The growing demand for increased food safety and superior quality has accelerated the introduction of new intelligent packaging strategies. Notably, packaging solutions based on nanotechnology have garnered significant attention from consumers across a wide spectrum of food product categories. These innovative approaches play a fundamental role in minimizing food waste and loss, owing to their potent antimicrobial properties, reinforced antioxidant potential, and enhanced sensory acceptance. Furthermore, in addition to health and nutritional considerations, sensory quality and user-friendliness of food products continue to be of paramount importance to consumers. The inherent characteristics of food, which significantly influence consumer purchasing behavior, encompass attributes such as color, aroma, and external features, including packaging labels and cultural differentiations. At present, nanopackaging stands as a promising method for food preservation. However, the lack of consumer awareness regarding advanced and emerging food technologies constitutes a significant barrier to the widespread acceptance of this innovative approach. Given this situation, intensified efforts to enhance knowledge, awareness, and public confidence are imperative. Such efforts are instrumental in expediting the transition towards adopting more sustainable packaging solutions [11].

In recent years, the use of nanoparticles such as clay, TiO_2_, Ag, Zn, etc., has gained more attention due to their physicochemical properties and antimicrobial features compared to conventional polymers [12, 13]. Currently, the utilization of polymer nanocomposite films, such as nanocomposites containing clay nanoparticles, is on the rise. This trend is attributed to the enhanced thermal and mechanical resistance as well as optimized permeability properties exhibited by these nanocomposites in comparison to conventional polymers, even on a macro scale [14]. Furthermore, attention has been directed towards the capability of TiO_2_ as a photocatalyst in deactivating a broad range of microorganisms such as *Pseudomonas aeruginosa*, *Rhodotorula mucilaginosa*, *Pseudomonas* spp., and mesophilic bacteria [15, 16].

Numerous studies have been conducted on the beneficial and promising effects of nanotechnology in food packaging. For instance, the utilization of packaging films containing nanoclay/starch LDPE as a biodegradable film [17], the use of kaolin/silver in producing a film for packaging asparagus [18], a film containing clay/silver nanoparticles and TiO_2_ to enhance the shelf life of kiwifruit [19], a nanocomposite film containing clay nanoparticles for preserving plums [20], and the application of a nanocomposite containing clay and TiO_2_ nanoparticles to extend the shelf life of pears are noteworthy examples [21]. Zamindar et al. (2022), reported that the combined polypropylene containing nano-hydroxyapatite with a modified atmosphere preserved the physicochemical and microbial characteristics of cherry tomatoes [22]. Recently, Gvozdenko et al. [23] introduced a novel method for synthesizing copper oxide nanoparticles stabilized with gelatin, specifically designed for use in food packaging. Their research revealed that these gelatin-stabilized CuO nanoparticles exhibited fungicidal properties, leading to an extension in the shelf life of both strawberry and tomato fruits.

To the best of our knowledge, there have been no reports on the use of LDPE-based nanocomposite films containing nanoparticles like clay, TiO_2_, and clay/ TiO_2_ for post-harvest tomato preservation as a cost-effective active packaging film. The current study aims to synthesize and characterize nanocomposite packaging films containing clay and TiO_2_ alone or in combination, and to evaluate their effects on the physico-chemical properties, antioxidant enzymes, and fungal decay of tomato cv. Newton during cold storage.

### Material and methods

The materials used in this study included low-density polyethylene (LDPE LF0200) with a melt flow index (MFI) of 2 gmin^−1^ from Arak Petrochemical Company, Iran, maleic anhydride from Gharanikin Company, Iran, Cloisite 20A clay from Sothern Clay Product, United States, Anatase and Rutile TiO_2_ nanopowders with particle sizes ranging from 20 to 80 nm from NanoShell Company, United States, and glycerol from Majalli Company, Iran. Other chemical reagents utilized in this research were sourced from Merck Company, Germany.

### Nanocomposite film preparation

The melt blending method was employed for the preparation and fabrication of nanocomposite films. TiO_2_ nanoparticles in both Anatase and Rutile phases were used in equal proportions due to their synergistic antioxidant activity. TiO_2_ nanoparticles were mixed with 3% Cloisite 20A clay nanoparticles, and 3% maleic anhydride was added as a compatibilizer (to enhance the blending of LDPE and clay nanoparticles). Additionally, 0.5% glycerol was used. Then, LDPE granules were gradually added, and gentle stirring was carried out for 1 h. An extruder apparatus (Brabender, model DSE 20: Germany) was utilized for reactive melt mixing at a constant temperature of 140 °C. The central screw was fixed at 130 rpm. The extruded material was cooled to 25 °C and subsequently pelletized using a milling device. Next, using a film-blowing machine, standard polyethylene films and nanocomposite films with a thickness of 30 ± 3 µm were obtained. Four types of synthesized films were employed in this experiment: neat LDPE polyethylene film (PE), nanocomposite film containing 3% clay nanoparticles (PE-C), nanocomposite film containing 3% TiO_2_ nanoparticles (PE-T), and nanocomposite film containing 3% TiO_2_ and 3% clay nanoparticles (PE-CT). The selected percentage of nanoparticles was based on our previous studies.

In this study, a commercial tomato cultivar named "Newton" at the ripe red stage was utilized. This cultivar was obtained from a local greenhouse in Shahrood, Iran. Immediately after collection, the samples were transferred to the laboratory for treatment applications.

Following the removal of unhealthy and contaminated fruits, uniformly sized and healthy fruits were washed three times with sterilized distilled water to eliminate field contaminants. They were dried at room temperature, and approximately 450 g of tomato fruits were packed in four different films were made as bags of 12 × 30 cm. Unpacked fruits placed on uncovered plastic trays were used as the control. The unpacked and packed fruits were stored in a refrigerator at a temperature of 4 ± 2° C and a humidity of 90% for 42 days under fluorescent light lamps (FL20S, Toshiba Co, Tokyo). The UVA light intensity on the surface of tomato fruits was approximately 0.05 mWcm^−2^, measured using a UVA-400 radiometer (S-365 UV-sensor, Iuchi, Osaka, Japan). Sampling was conducted on both the packed and control tomato fruits every seven days, over a six storage interval. Physicochemical attributes were evaluated, including ethylene production, weight loss percentage, tissue firmness, (total soluble solids) TSS, titratable acidity (TA), pH, electrolyte leakage, malondialdehyde (MDA) content, and the activity of antioxidant enzymes, including catalase (CAT) and ascorbate peroxidase (APX), along with microbial population assessment.

### Mechanical properties of films

To evaluate the mechanical properties of films, a film testing apparatus (Instron 6025, UK) was employed. Six specimens were measured for each sample, with dimensions of 5 × 2.5 cm and a thickness of 30 ± 3 µm. Prior to testing, the samples were conditioned at 23 °C and 50% RH for 2 days. The distance between the grips of the device was set at 50 mm. Evaluation of mechanical properties included Young's modulus, stress, and strain at break were determined under a temperature of 25 °C and relative humidity of 30%, with a crosshead speed of 50 mm per minute using a tensile tester, following ISO 527–2 standard [24].

### Permeability assessment of nanocomposite films

Permeability of O_2_ and CO_2_ was respectively measured using the OX-TRAN 2/21 and PERMATRAN-C Model 4/41 devices at a temperature of 25 °C. The values of O_2_ and CO_2_ permeation were expressed as cc-mil m^−2^ day^−1^. Water vapor permeability (WVP) was evaluated using the L80-5000 tester (Amical) at a temperature of 25 °C. WVP values were presented as g mm kPa^−1^ h^−1^ m^−2^.

### Contact angle measurements

The contact angles of nanocomposite films were assessed utilizing a contact angle meter (Pendant drop contact angle, CA-ES10, Fars EOR Tech CO., Iran) employing the sessile drop method. A 4 µL droplet of deionized water was carefully placed on the surface of the film. The dynamic contact angle was ascertained by fitting a mathematical equation to the water droplet's shape and determining the tangent between the water drop and solid film. Contact angle measurements were conducted in triplicate for all samples.

### TEM

Micrographs were obtained using a PHILIPS EM-2085 transmission electron microscope under the control of a microprocessor, operating at 100 kV.

### Headspace gas composition

Before unsealing the tomato fruit bags, headspace gas composition analyses were performed employing a Checkmate 990 gas analyzer (PBI Dansensor, DK-4100 Ringsted, Denmark). The gas sample extracted from the package headspace for analysis was approximately 10 cm^3^. To enhance measurement accuracy, a single determination of the headspace gas composition was conducted for each package. The findings of the O_2_ and CO_2_ assays were expressed as percentages (%).

### Ethylene production

The assessment of ethylene produced by tomato fruits during storage was conducted following the method described by Pretel et al. [25]. In each trial, control and packaged fruits, after opening the fruit bags were placed in a 1000 ml plastic container equipped with a septum for 3 h at a temperature of 20 °C. Subsequently, 10 ml of gas from the headspace was withdrawn and transferred to a vacuumed glass vial. Then, 1 ml of the gas volume from the glass vial was injected into a chromatography instrument equipped with an HP-5 µs column (Agilent, United States of America). The column temperature was set at 55 °C, and the injector and detector temperatures were set at 110 °C. The amount of ethylene produced by tomato fruits was expressed in ng kg^−1^ s^−1^.

### Weight loss percentage

For the measurement of weight loss percentage, samples were weighed at the beginning and the end of a specified storage period. The weight loss percentage was calculated using the following formula (Eq. 1):

1 $$\mathrm{Weight}\,\mathrm{loss}\,\mathrm{percentage}\,=\,100\times\,((\mathrm{Initial}\,\mathrm{Weight}-\mathrm{Final}\,\mathrm{Weight})/\,\mathrm{Initial}\,\mathrm{Weight})$$

### Firmness

To measure the tissue firmness was measured as puncture force in tomato fruit using a handheld digital fruit hardness tester (model 41050, Germany) with a 5-mm cylindrical probe. The test was performed at 2 points by taking skin-off measurements on both sides of the fruit in three replications, and the results were expressed in N.

### TSS, TA and pH

TSS was measured using a handheld refractometer the data were expressed as °Brix.

pH measurement was conducted using 50 g of tomato fruit samples. These samples were homogenized, and subsequently, the homogenized materials underwent centrifugation at 10,000 × g for 20 min. The resulting clear supernatant was utilized for pH measurement with a pH meter (WTW model, Germany).

For the measurement of TA, 1 ml of the supernatant was diluted with 20 ml of distilled water. The diluted solution was titrated with 0.1 N sodium hydroxide solution to pH 2.8, and the dominant organic acid (citric acid) content was calculated.

### Lipid peroxidation and electrolyte leakage

MDA content in tomato fruit was measured following the method of Luo et al. [26]. Tissue samples (2 g) were frozen in liquid nitrogen, and rapidly ground, and the extraction process was done in 5 ml 10% (w/v) trichloroacetic acid (TCA). The homogenized samples were then centrifuged at 9000 × g for 20 min, and 2 ml of supernatant was mixed with 2 ml 10% (w/v) TCA containing 0.5% (w/v) thiobarbituric acid (TBA) solution. After heating the mixture at 100 °C for 20 min, it was quickly cooled and centrifuged at 10,000 × g for 10 min. The absorbance at 450, 532, and 600 nm was measured using a Unico spectrophotometer (model 2150, China), and the MDA content was calculated according to the formula (Eq. 2):

2 $$6.45\times({\mathrm{OD}}_{532}-{\mathrm{OD}}_{600})\,-\,0.56\times\,{\mathrm{OD}}_{450}.$$

The electrolyte leakage was determined using the method described by Huang et al. [27]. Disks with a diameter of 10 mm with a cork borer were taken from the fruits of each sample. A total of 10 disks were incubated in 15 ml of distilled water at a temperature of 25 °C and shaken for 30 min, (EC1). An additional set of 10 disks was subjected to distilled water at a temperature of 100 °C for 25 min, utilizing a water bath (EC_2_). Subsequently, the electrolyte leakage was assessed using an EC meter (EC-400L), following the formula provided as (Eq. 3): Electrolyte leakage (%) = (EC2)/(EC1) × 100 (3).

### Antioxidant enzyme analysis

CAT activity was evaluated utilizing the procedure established by Wang et al. [28], with some modifications. The experimental protocol involved the utilization of 1 ml of sodium phosphate buffer (50 mM, pH 7.0), 1 ml of 40 mM H_2_O_2_, and 1 ml of enzyme extract. A rate of change in optical density at 240 nm of 0.01 per min was designated as one unit of CAT activity. The obtained outcomes were subsequently quantified as units per gram of fresh weight (U g^−1^ FW).

The enzymatic activity of APX was assessed using the methodology introduced by Sofo et al. [29], with slight modifications. For the enzyme extract, 1 g of the sample was homogenized in 2 ml of 100 mM phosphate buffer. The reaction mixture consisted of 825 µl of 100 mM potassium phosphate buffer, 25 µl of 1 mM ascorbic acid solution, 52 µl of 5.2 mM H_2_O_2_ solution, and 100 µl of enzyme extract. Initially, the mixture was transferred into a 3-ml quartz cuvette, and the absorbance was measured at a wavelength of 290 nm using a spectrophotometer (Unico, model 2150, China).

### Mold population assessment

Antimicrobial analysis of mold population was done as described by Del Nobile et al. [30]. Every 7 days, around 50 g of tomato segments (which were sliced both vertically and horizontally, encompassing a portion of the peel) were arbitrarily chosen. These segments were then combined with 180 ml of saline solution (0.9%) and subjected to agitation within a stomacher bag for 120 s. Subsequent decimal dilutions were carried out using sterile saline solution, following which 0.1 ml of both the undiluted solution and the diluted solutions were cultured on Sabouraud dextrose agar, and mold colonies were counted and reported as logarithmic colony-forming units (log CFU/g).

### Statistical analysis

The experiment was conducted as a completely randomized design (CRD) in a bi-factorial model (storage time × treatments) with triplicate. Data were analyzed using SAS software version 9.1 and visualized using Excel 2013. Mean comparisons were performed using Duncan's test at a significance level of *p* < 0.05. The values are reported as mean ± standard deviation.

## Results

### Nanocomposite film characteristics

#### Mechanical properties of films

The possession of appropriate mechanical strength to enhance structural stability and the permeability properties of the film holds significant importance [19]. To investigate the performance of clay and TiO_2_ nanoparticles, the mechanical and permeability properties of nanocomposite films were evaluated. The results revealed that Young's modulus of PE-C, PE-T, and PE-CT films increased by approximately 34%, 24%, and 58%, respectively, compared to the neat polyethylene film (Table 1).

**Table 1:** Mechanical properties of nano-composite packaging and LDPE films

Films	Young’s modulus (Pa)	Stress at break (MPa)	Strain at break (%)
PE	89.53 ± 4.2^c♦^	10.4 ± 0.32^c^	128.6 ± 12.1^b^
PE-C	120.2 ± 6.2	12.2 ± 0. 42^a^	116.3 ± 14.2^c^
PE-T	111.32 ± 5.7^b^	11.2 ± 0. 36^b^	136.1 ± 21.1^a^
PE-TC	141.31 ± 8.3^a^	12.8 ± 0.51^a^	126.31 ± 9.2^b^

^♦^Means with the same letters within column are not significantly (*p *> 0.05) different according to the Duncan´s multiple range tests. PE (neat LDPE), PE-C (nanocomposite film containing 3% clay nanoparticles), PE-T (nanocomposite film containing 3% TiO_2_ nanoparticles), PE-CT (nanocomposite film containing 3% TiO_2_ and 3% clay nanoparticles)

### CO_2_, O_2_ and water vapor permeability of films

With the addition of clay content in the nanocomposite films, the permeability of these films to CO_2_ exhibited a significant decrease, so that the permeability of PE-C film (25,521 cc-mil m^2^ day^−1^) was reduced by approximately 32% compared to PE film permeability (38,012 cc-mil m^2^ day^−1^) (Table 2). The difference in permeability of the nanocomposite films to O_2_ was significantly pronounced with the addition of clay nanoparticles. The resulting film from the PE-C nanocomposite exhibited the lowest permeability, measuring 9321 cc-mil m^2^ day^−1^, whereas the PE film showed the highest O_2_ permeability, measuring 19,203.04 cc-mil m^2^ day^−1^. The addition of 3% clay nanoparticles to the LDPE matrix led to a reduction in O_2_ permeability by 51% compared to neat LDPE. The inclusion of TiO_2_ nanoparticles in the PE-T nanocomposite film did not show a considerable impact on reducing permeability. The difference in permeability between the PE-C and PE-CT nanocomposite films compared to the PE film against water vapor was also observed, and the results indicated that the addition of 3% clay to the nanocomposite films resulted in a reduction of water vapor permeability by about 5% and 12% for PE-C and PE-CT films, respectively, in comparison to PE film (Table 2).

**Table 2:** Barrier properties of nanocomposite packaging and LDPE films

Films	O_2_ transmission (cc-mil m^−2^ day^−1^)	CO_2_ transmission (cc-mil m^−2^ day^−1^)	Water vapor permeability (g mm kPa^−1^ h^−1^ m^−2^)
PE	19,203 ± 451.2^a♦^	38,012.2 ± 1320^a^	2.88 ± 0.03^a^
PE-C	9321 ± 981.2^c^	25,521.01 ± 1151^c^	2.71 ± 0.02 ^b^
PE-T	13,908 ± 1231^b^	35,921.06 ± 922^b^	2.78 ± 0.01^b^
PE-TC	10,441 ± 652^c^	27,912 ± 1020^c^	2.62 ± 0.04^c^

Data were represented as means ± the standard deviation (*n* = 3). ^♦^Means with the same letters within column are not significantly (*P* > 0.05) different according to the Duncan´s multiple range tests. PE (neat LDPE), PE-C (nanocomposite film containing 3% clay nanoparticles), PE-T (nanocomposite film containing 3% TiO_2_ nanoparticles), PE-CT (nanocomposite film containing 3% TiO_2_ and 3% clay nanoparticles)

### Contact angle

**Fig. 1:**
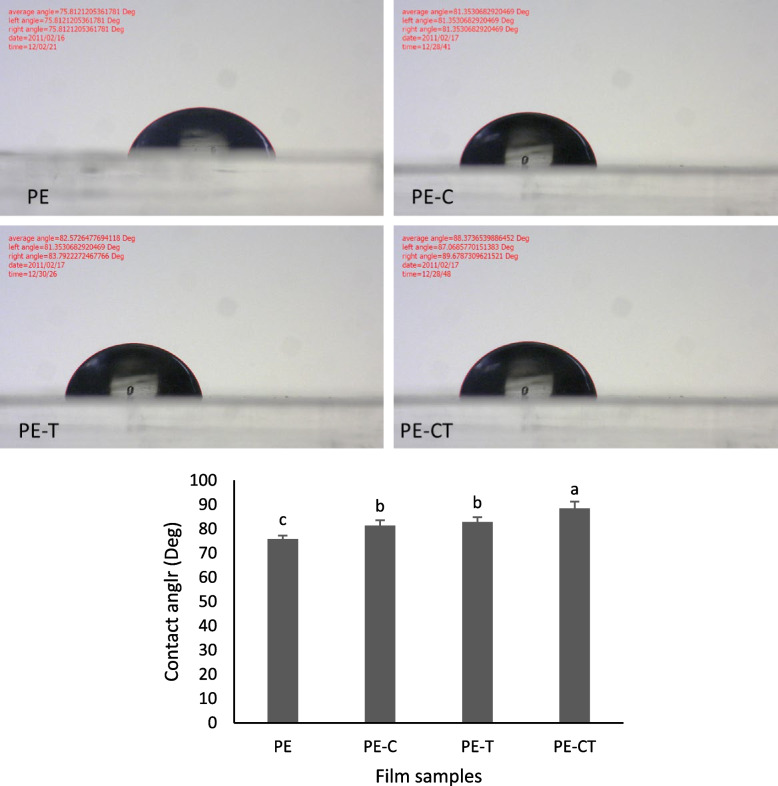
Effect of clay and TiO_2_ on the contact angle of nanocomposite films. Data were represented as means ± the standard deviation (*n* = 3). Any two means in the same column followed by the same letter are not significantly *(P* > 0.05) different according to the Duncan’s multiple range test. PE (neat LDPE), PE-C (nanocomposite film containing 3% clay nanoparticles), PE-T (nanocomposite film containing 3% TiO_2_ nanoparticles), PE-CT (nanocomposite film containing 3% TiO_2_ and 3% clay nanoparticles), control (unpacked tomato fruits)

Water contact angle results are presented in Fig. 1. The water contact angle of PE film was 75.81°. By the addition of 3% clay and TiO_2_ nanoparticles, the contact angle increased to 81.35° and 82.75° in PE-T and PE-C nanocomposite films, respectively. The highest value of 88.37°, was obtained in PE-CT nanocomposite film in comparison to PE film.

### TEM

**Fig. 2:**
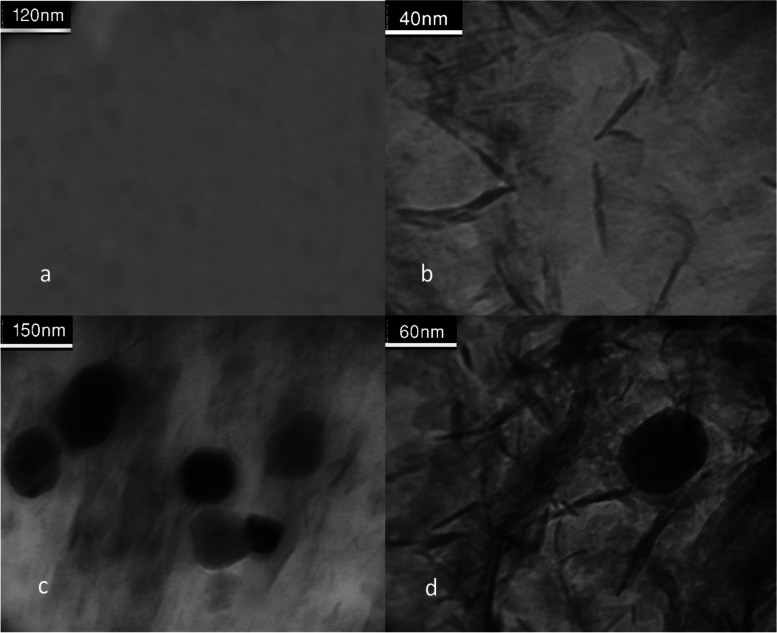
Transmission electron micrographs of nanocomposites: **a** PE, **b** PE-C, **c** PE-T, and **d** PE-CT films

Nanoparticle dispersion plays a crucial role during nanocomposite film preparation. Agglomerations of additives within the polymeric matrix are believed to disrupt film integrity, leading to decreased plasticity and quality [31]. In Fig. 2, TEM micrographs depict various samples: PE, exfoliated PE-C nanocomposite, PE-T nanocomposite, and PE-CT nanocomposites. The polymeric matrix is denoted by clear or white regions (Fig. 2a), while nanoparticles are depicted in dark areas. Notably, Fig. 2b reveals well-distributed nanoclay and effective nanoparticle exfoliation within the PE-C film. Figure 2c illustrates the relatively good dispersion of Anatase and Rutile in PE-T, while Fig. 2d reveals the exfoliation of clay nanoparticles with two TiO_2_ nanoparticles in PE-CT.

### Headspace gas composition

**Fig. 3:**
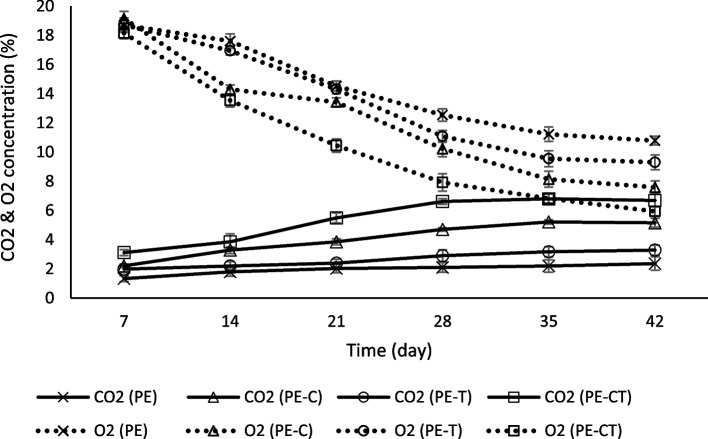
Effects of nanocomposite packaging films on headspace gas composition (CO_2_ and O_2_) of packed tomato fruit during storage at 4 °C. Data were represented as means ± the standard deviation (*n* = 3). PE (neat LDPE), PE-C (nanocomposite film containing 3% clay nanoparticles), PE-T (nanocomposite film containing 3% TiO_2_ nanoparticles), PE-CT (nanocomposite film containing 3% TiO_2_ and 3% clay nanoparticles)

Figures 3 illustrate the O_2_ and CO_2_ concentrations in the headspace of the package during storage. Notably, nanocomposite films, particularly PE-CT and PE-C, exhibited a significant reduction in O_2_ and an increase in CO_2_ levels in the headspace of the packed fruits. Packed fruits in PE-CT and PE-C recorded higher CO_2_ and lower O_2_ levels compared to those in PE-T and PE. After 28 days of storage, the O_2_ content in PE-CT and PE-C packages decreased by 7.92% and 10.23%, respectively, which was significantly lower than that observed in PE-T and PE packages (11.08% and 12.54%, respectively). At the end of the storage time, the O_2_ concentration in PE-CT and PE-C packages remained significantly lower than in PE-T and PE packages. Conversely, there was an opposite trend in CO_2_ content. In the initial 28 days, CO_2_ content surged to 6.61% and 4.7% in PE-CT and PE-C packages, respectively, while it only reached 2.9% and 2.1% in PE-T and PE packages, respectively. Equilibrium-modified atmospheres were almost established after 28 days for all treated fruits during storage (Fig. 3)

### Ethylene production

**Fig. 4:**
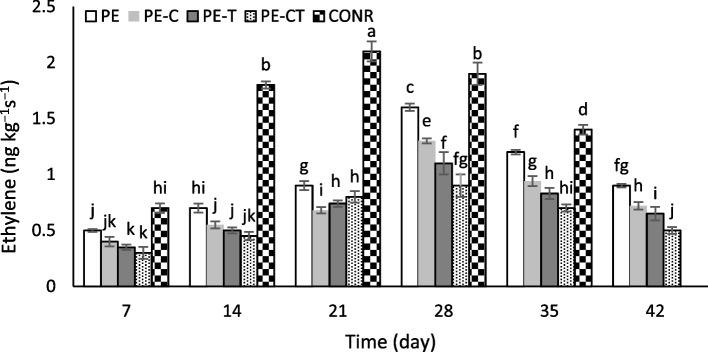
Effects of nanocomposite packaging films on ethylene production of tomato fruits during 42 days storage at 4 °C. Data were represented as means ± the standard deviation (*n* = 3). PE (neat LDPE), PE-C (nanocomposite film containing 3% clay nanoparticles), PE-T (nanocomposite film containing 3% TiO2 nanoparticles), PE-CT (nanocomposite film containing 3% TiO2 and 3% clay nanoparticles), control (unpacked tomato fruits)

The mitigation of ethylene accumulation in packed fruits poses a challenging endeavor, even when subjected to low-temperature storage conditions. A remarkable increment in ethylene emission was discerned in unpacked fruit on the 12th day while the peak of ethylene release in packed fruits was observed on the 35th day (Fig. 4). Packed fruit in PE-C, PE-T and PE-CT films exhibited solely marginal rates of ethylene generation. The ethylene concentration in PE film (0.9 ng kg^−1^ s^−^1) showed an increase about of 1.8-fold compared to PE-CT film (0.5 ng kg^−1^ s^−1^) on the final day of the study.

### Weight loss

**Fig. 5:**
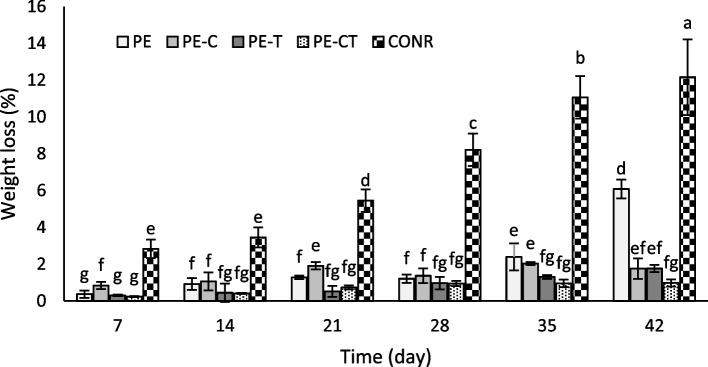
Effects of nanocomposite packaging films on weight loss of tomato fruits during 42 days storage at 4 °C. Data were represented as means ± the standard deviation (n = 3). PE (neat LDPE), PE-C (nanocomposite film containing 3% clay nanoparticles), PE-T (nanocomposite film containing 3% TiO_2_ nanoparticles), PE-CT (nanocomposite film containing 3% TiO_2_ and 3% clay nanoparticles), control (unpacked tomato fruits)

The progressive weight loss trend in both packed and unpacked tomatoes is observed passing the time, as depicted in Fig. 5, the weight reduction differences until day 28 among packed tomatoes using nanocomposite and PE films did not exhibit statistically significant differences. However, after 28 days until the end of the storage period, notable differences in weight loss were evident across various film types. The greatest weight loss occurred in unpacked tomatoes, followed by tomatoes packaged with PE films, with weight losses of 12.15% and 6.08% after 42 days, respectively. The least weight loss was observed in tomatoes packed with PE-CT films, indicating a weight loss of 0.98%.

## Firmness

**Fig. 6:**
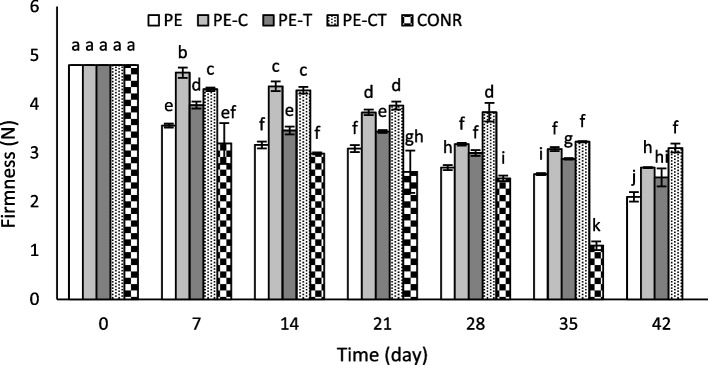
Effects of nanocomposite packaging films on firmness of tomato fruits during 42 days storage at 4 °C. Data were represented as means ± the standard deviation (*n* = 3). PE (neat LDPE), PE-C (nanocomposite film containing 3% clay nanoparticles), PE-T (nanocomposite film containing 3% TiO_2_ nanoparticles), PE-CT (nanocomposite film containing 3% TiO_2_ and 3% clay nanoparticles), control (unpacked tomato fruits)

The effect of different packaging films on the fruit tissue firmness of tomatoes throughout the storage period is illustrated in Fig. 6. Regardless of the treatment type, the firmness level decreased in all fruits from the beginning of the experiment (4.8 N) to the end of the storage period. The reduction in firmness over the 35 days from the start of the experiment was approximately 51% for fruits packed with PE and 32% for fruits packaged with PE-CT while the firmness reduction was notably higher by 82% for unpacked fruits. At the end of the experiment, the firmness level in the PE treatment decreased to 2.1 N. The highest firmness values were observed in the PE-CT and PE-C treatments, approximately 3.1 N and 2.7 N, respectively.

### The effect of nanocomposite film on TSS, TA and pH

Measurement of TSS, TA, and pH in packed fruits was feasible throughout the experiment due to the presence of sufficient water and the preservation of tissue moisture. However, in unpacked fruits, measurements were attainable only until day 35 due to weight loss, wrinkling, and spoilage. At the beginning of the experiment (day zero), the TA and pH were 26.5% and 4.3, respectively (Table 3). In all treatments, TA decreased while pH increased over time. Evaluation of pH levels during the storage period of tomato fruits revealed significant differences among various treatments. The highest pH value (4.78) was observed in unpacked fruits after 35 days, whereas these values after 48 days for the PE, PE-T, PE-C, and PE-CT treatments were 4.8, 4.76, 4.64, and 4.63, respectively. Changes in TA became noticeable in unpacked fruits from day 7 onwards, while noticeable changes in packed fruits were evident from day 21 onwards, decreasing steadily until the end of the storage period. The highest protection against TA reduction in packed fruits was observed in the PE-CT film (14.4%), and the most significant changes occurred in packed fruits using the PE film (10.66%) on day 48, and unpacked fruits (12.58%) on day 35. Changes in TSS also followed a similar pattern to pH changes.

**Table 3:** Effect of nanocomposite packaging films on pH, titrable acidity (TA) and total soluble solids (TSS) of tomato fruits during cold storage period

Treatments	Storage time (Days)						
	0	7	14	21	28	35	42
*pH*							
PE	4.3^d♦^	4.59 ± 0.01^ cd^	4.7 ± 0.03^b^	4.66 ± 0.014^bc^	4.66 ± 0.028^bc^	4.67 ± 0.014^bc^	4.81 ± 0.007^b^
PE-C	4.3^d^	4.54 ± 0.02^c^	4.56 ± 0.02^c^	4.57 ± 0.013^c^	4.65 ± 0.007^c^	4.63 ± 0.006^c^	4.64 ± 0.02^c^
PE-T	4.3^d^	4.53 ± 0.016^c^	4.55 ± 0.028^c^	4.66 ± 0.031^bc^	4.7 ± 0.035^bc^	4.71 ± 0.017^bc^	4.76 ± 0.028^b^
PE-TC	4.3^d^	4.47 ± 0.03^c^	4.56 ± 0.042^c^	4.58 ± 0.014^c^	4.58 ± 0.02^c^	4.61 ± 0.014^c^	4.63 ± 0.04^c^
Control	4.3^d^	4.54 ± 0.035^c^	4.58 ± 0.028^c^	4.73 ± 0.024^b^	4.76 ± 0.021^b^	4.9 ± 0.045^a^	-
*TA (%)*							
PE	26.5^a^	23.82 ± 0.93^bc^	22.70 ± 0.48^bc^	18.83 ± 0.80^d^	13.58 ± 0.39^ef^	12.71 ± 0.35^f^	10.66 ± 0.90^f^
PE-C	26.5^a^	24.4 ± 0.89^b^	23.3 ± 0.79^bc^	15.90 ± 0.67^de^	15.39 ± 0.74^de^	14.27 ± 0.42^e^	13.49 ± 0.44^e^
PE-T	26.5^a^	22.98 ± 0.7b^c^	21.45 ± 0.^8c^	17.24 ± 0.45^d^	15.78 ± 0.68^de^	14.02 ± 0.3^e^	12.92 ± 0.9^g^
PE-TC	26.5^a^	25.32 ± 0.16^b^	24.39 ± 0.55^b^	22.16 ± 0.57^bc^	15.81 ± 0.84^de^	16.61 ± 0.34^de^	14.40 ± 0.70^de^
Control	26.5^a^	21.2 ± 0.89^c^	15.04 ± 0.74^e^	13.86 ± 0.9^ g^	11.31 ± 0.82^ g^	12.58 ± 0.43^f^	-
*TSS (°Brix)*							
PE	2.9^ g^	3.3 ± 0.07^f^	3.95 ± 0. 19^d^	4.15 ± 0.07^cd^	4.4 ± 0.07^c^	4.85 ± 0.14^b^	4.9 ± 0.1^b^
PE-C	2.9^ g^	3.75 ± 0.12^d^	3.8 ± 0.07^d^	3.95 ± 0.15^d^	4.05 ± 0.14^d^	4.09 ± 0.08^d^	4.15 ± 0.13^d^
PE-T	2.9^ g^	3.70 ± 0. 14^d^	3.85 ± 0.07^d^	4.05 ± 0.07^d^	4.1 ± 0.08^d^	4.18 ± 0.1^d^	4.3 ± 0.12^cd^
PE-TC	2.9^ g^	3.05 ± 0.2^f^	3.2 ± 0.14^f^	3.45 ± 0.1^e^	3.85 ± 0.18^de^	3.9 ± 0.06^de^	4 ± 0.08^d^
Control	2.9^ g^	3.9 ± 0.12^d^	4.4 ± 0.15^c^	4.45 ± 0.09^c^	4.85 ± 0.06^b^	5.2 ± 0.05^a^	-

Data were represented as means ± the standard deviation (*n* = 3). ^♦^Means with the same letters within column are not significantly (*P* > 0.05) different according to the Duncan’s multiple range tests. PE (neat LDPE), PE-C (nanocomposite film containing 3% clay nanoparticles), PE-T (nanocomposite film containing 3% TiO_2_ nanoparticles), PE-CT (nanocomposite film containing 3% TiO_2_ and 3% clay nanoparticles), control (unpacked tomato fruits)

### Membrane stability and membrane lipid peroxidation

**Fig. 7:**
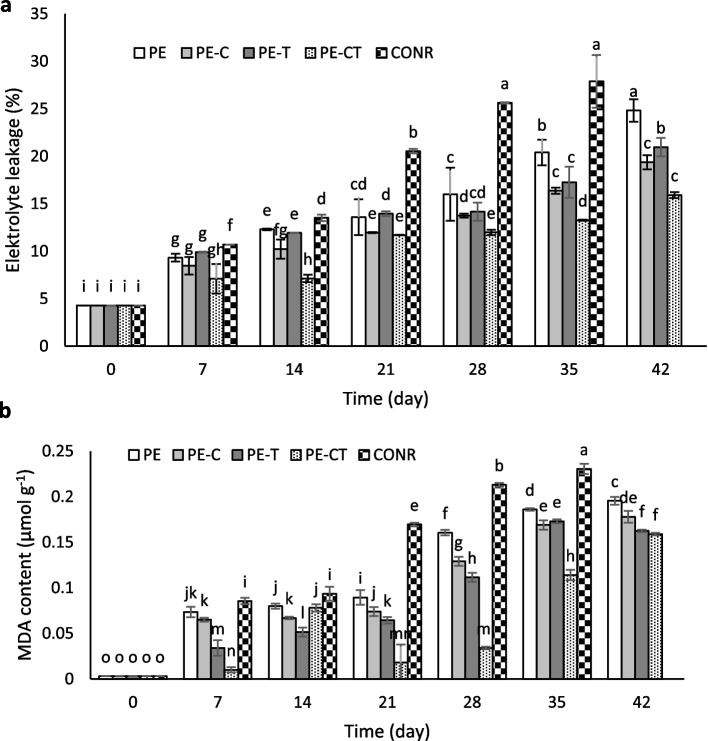
Effects of nanocomposite packaging films on electrolyte leakage (**a**) and lipid peroxidation (**b**) of tomato fruits during 42 days storage at 4 °C. Data were represented as means ± the standard deviation (*n* = 3). PE (neat LDPE), PE-C (nanocomposite film containing 3% clay nanoparticles), PE-T (nanocomposite film containing 3% TiO_2_ nanoparticles), PE-CT (nanocomposite film containing 3% TiO_2_ and 3% clay nanoparticles), control (unpacked tomato fruits)

As illustrated in Fig. 7, both electrolyte leakage and MDA content exhibited similar trends, wherein these values increased over time in all packed and unpacked fruits. On day 35, the highest electrolyte leakage was observed in unpacked fruits (27.89%) and packed fruit with PE film (20.40%), while the lowest electrolyte leakage was found in packed fruits with a film containing both clay nanoparticles and TiO_2_ (PE-CT), amounting to 15.92% at the end of the storage time. Notably, electrolyte leakage in packed fruits within the PE (20.96%) was significantly higher compared to the nanocomposite film treatments (Fig. 7a).

The MDA content steadily increased throughout the storage period in both unpacked and packed tomatoes (Fig. 7b). Concurrent utilization of clay nanoparticles and TiO_2_ effectively led to a reduction in MDA production in fruits packed with the PE-CT film, resulting in an MDA content of approximately 0.15 µmol g^−1^ fresh weight, in contrast to 0.19 µmol g^−1^ for PE film packaging, representing a decrease of about 26%.

### Effect of nanocomposite films on antioxidant enzymes

**Fig. 8:**
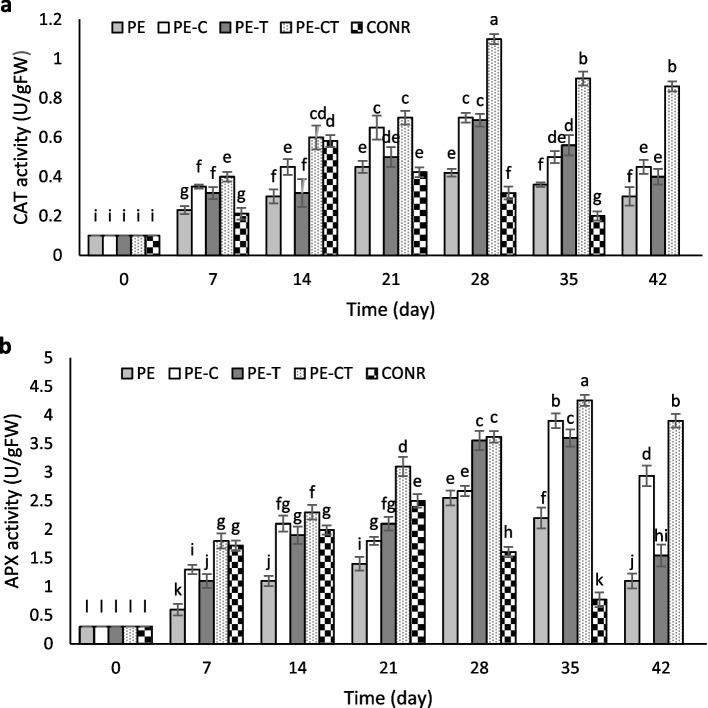
Effects of nanocomposite packaging films on CAT (**a**) and APX (**b**) activity of tomato fruits during 42 days storage at 4 °C. Data were represented as means ± the standard

As shown in Fig. 8, the CAT enzyme activity levels increased to 1.1, 0.68, and 0.70 U g^−1^ FW, respectively, in fruits packed with PE-CT, PE-T, and PE-C films on day 35, followed by a decrease to 0.86, 0.40, and 0.45 U g^−1^ FW at the end of the experimental period (Fig. 8a). These findings indicated that the highest CAT enzyme activity was observed in fruits packed with nanocomposite films throughout the storage time, whereas the enzyme activity decreased in fruits packed with PE films and unpacked fruits on days 21 and 14, respectively. The APX enzyme activity showed minimal differences between unpacked and packed fruits until day 7, but a significant increase was observed from day 7 to the end of the experiment in fruits packed with nanocomposite films (Fig. 8b). APX activity decreased in both unpacked and PE-packed fruits on days 21 and 28, respectively. The highest activity of this enzyme at the end of the storage period was observed in fruits packed with PE-CT and PE-C films at 3.9 and 2.9 U g^−1^ FW, respectively, while it was 1.1 U g^−1^ FW in fruits packed with PE film.

### Effect of nanocomposite films on fungal decay

**Fig. 9:**
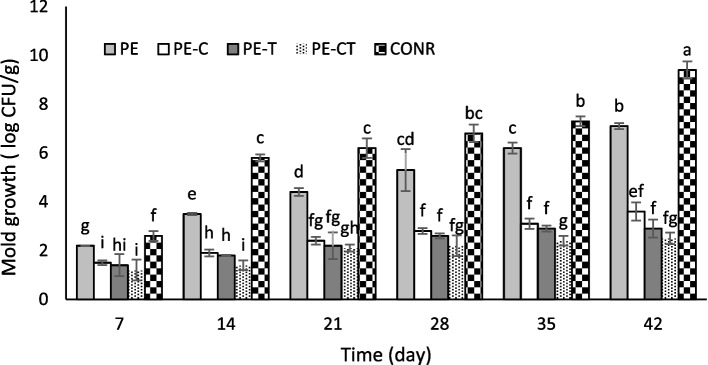
Effects of nanocomposite packaging films on mold growth in tomato fruits during 42 days storage at 4 °C. Data were represented as means ± the standard deviation (*n* = 3). PE (neat LDPE), PE-C (nanocomposite film containing 3% clay nanoparticles), PE-T (nanocomposite film containing 3% TiO_2_ nanoparticles), PE-CT (nanocomposite film containing 3% TiO_2_ and 3% clay nanoparticles), control (unpacked tomato fruits)

Microbiological evaluation of stored tomato fruits during storage at 4 °C was conducted by enumerating the total fungal colonies formed (Fig. 9). After 7 days, the formed fungal colonies in tomato fruits ranged from approximately 1.2 to 2.6 log CFU/g. Over time, the difference in formed fungal colonies became more pronounced between unpacked fruits, PE-packed fruits, and nanocomposite film-packed fruits.

**Fig. 10:**
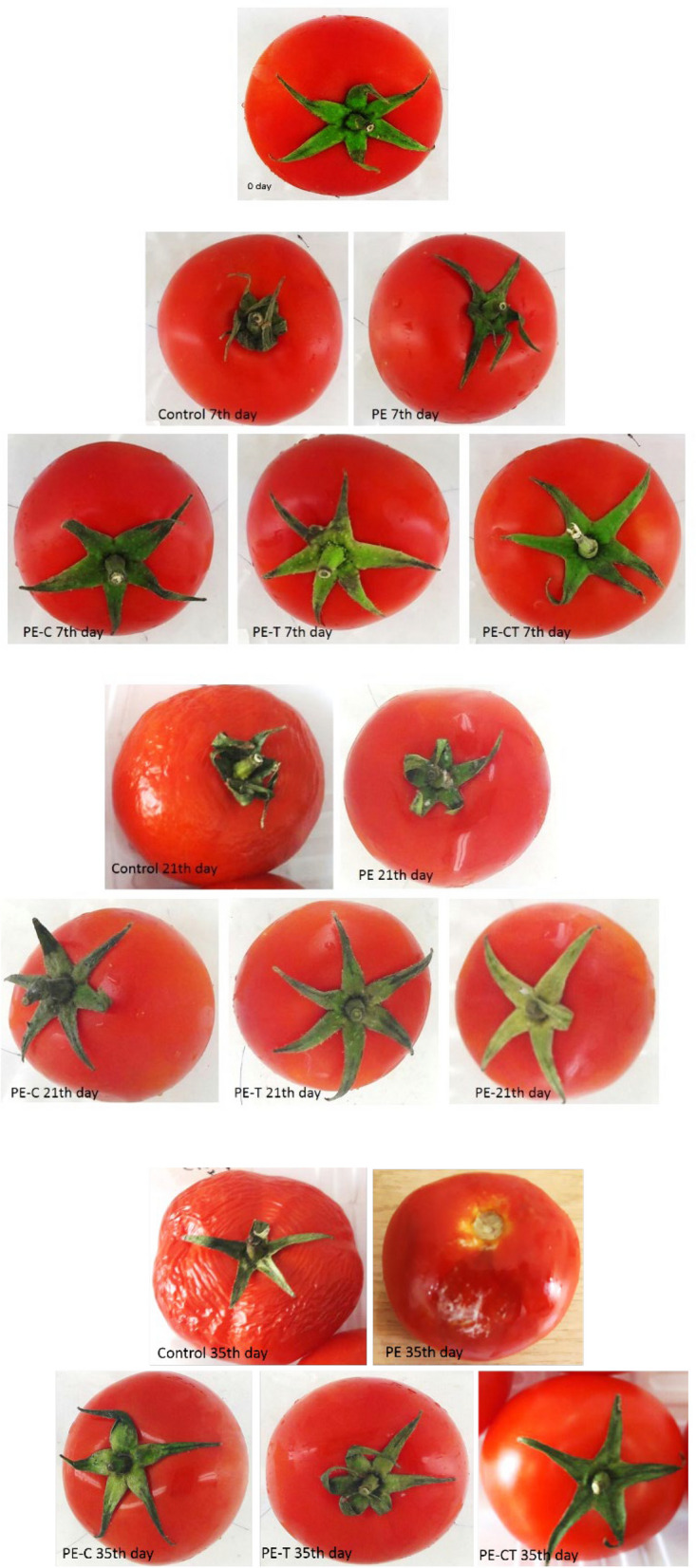
The appearance of tomato fruit during 42 days storage at 4 °C. PE (neat LDPE), PE-C (nanocomposite film containing 3% clay nanoparticles), PE-T (nanocomposite film containing 3% TiO_2_ nanoparticles), PE-CT (nanocomposite film containing 3% TiO_2_ and 3% clay nanoparticles), control (unpacked tomato fruits)

Although the increase in fungal population was also observed in fruits packed with PE-CT and PE-T, it was comparatively lower than that in PE-C packed fruits. At the end of the experiment, the highest fungal population was recorded in unpacked fruits (9.4 log CFU/g) and PE-packaged fruits (7.1 log CFU/g), while the least fungal growth was observed in fruits packed with PE-CT and PE-T films (2.5 and 2.9 log CFU/g, respectively). The appearance of tomato fruits during storage time was presented in Fig. 10.

## Discussion

As observed in Table 1, the addition of 3% clay (closite 20 A) nanoparticles into the LDPE matrix improved the strength of the nanocomposites compared to PE, confirming the reinforcing effect of nanoparticles in the polymer matrix, consistent with previous research findings [32, 33]. Furthermore, it can be inferred that by adding TiO_2_ nanoparticles as fillers, Young's modulus improved. The highest Young's modulus was achieved in the PE-CT film, where a synergistic interaction between clay and TiO_2_ nanoparticles was evident, as also reported in earlier studies using 5% clay and 3% TiO_2_ [34, 35].

The tensile strength of clay, TiO_2_, and the mixture of clay and TiO_2_ in PE-CT films with a 3% clay and 3% TiO_2_ blend exhibited an increase compared to PE. This increase can be attributed to the proper distribution of nano-fillers within the polymer matrix, resulting in improved tensile strength in the nanocomposite films. According to Liang et al.'s findings (2005), the distribution of clay nanoparticles with a higher aspect ratio in the polymer matrix can enhance stress resistance [36]. The stronger interaction between the layers of clay and the molecular polymer resulting from a larger contact area leads to a more effective constraint on the polymer's movement. The relative increase in tensile strength in the PE-CT nanocomposite may be attributed to achieving a homogeneous structure of clay and TiO_2_ nanoparticles with polyethylene polymer. According to Golebiewski et al. (2008), under increased loading of nanoparticles as fillers, the nanoparticles transform into a microlayer arrangement due to the adhesion of silicate layers [37].

In contrast to stress at peak, strain at peak in the clay-nanocomposite films decreased compared to the TiO_2_-nanocomposite films. One of the reasons for this reduction could be the relatively low content of compatibilizer (3%) during nanocomposite production, as Golebiewski et al. [37] reported the attainment of the highest strain at peak with a compatibilizer content of 15%.

Nanoparticles of clay act as physical barriers within nanocomposite films, leading to a decrease in permeability to O_2_, CO_2_, and water vapor. These nanoparticles hinder the movement of gases within the film layers, thereby slowing the gas flow rate and elongating the path that gas molecules must traverse through the film layer. In contrast to the direct path through films of PE without nanoparticles, which occurs due to the presence of small clay platelets, the introduction of nanoparticles in nanocomposite films results in a zigzag and tortuous gas diffusion path [32]. In films lacking nanoparticles, no barrier impedes gas movement, hence no reduction in permeability is observed. The findings of this study are consistent with the assertions of Pereira-de-Abreu et al. [32]. They noted that the addition of clay nanoparticles, specifically Closite 15A, to polypropylene and polyethylene polymers decreased their permeabilities to O_2_ by 22% and 12.5%, respectively compared to neat LDPE while, our results also confirmed the decrease in the permeability of the nanocomposite film against water vapor. The decrease in permeability of polyethylene nanocomposite films to O_2_, CO_2_, and water vapor can also be attributed to the nature of the polymer itself. LDPE exhibits relatively good resistance to water vapor permeability [12]. In PE-C and PE-CT films, the addition of clay nanoparticles further reduces WVP. Furthermore, while the radius of the molecule of O_2_ and water molecules are equivalent [38], their mechanisms of penetration as permeants in a polymer matrix are distinct due to their polarity differences. This polarity discrepancy results in water molecules unavoidably passing through the film layer in a clustered manner [32]. The findings of the current study align with a previous study conducted by Rim et al. [10], which investigated the impact of adding Cloisite 30B and Cloisite 20A on the water permeability of poly (lactic acid) nanocomposite film. It was observed that at a 5% weight addition of nanoparticles, there was a 15% increase in water permeability. Specifically, Cloisite 30B demonstrated a 5% reduction, while Cloisite 20A exhibited a more substantial reduction of 36%. The greater hydrophobicity of Cloisite 20A suggests that it may be more effective than Cloisite 30B. In summary, the reduction of gas permeability in nanocomposite films can enhance the capabilities of these films in extending the shelf life of packed food products.

The hydrophobicity of nanocomposite films plays a crucial role in influencing permeation flux through thin films, as assessed through contact angle measurements. Incorporating TiO_2_ nanoparticles decreased the hydrophilicity of neat PE films. The integration of TiO_2_ nanoparticles directly influences the surface roughness of the film. The resulting heightened roughness emerges as a significant contributor to the observed reduction in hydrophilicity [39]. Our results are consistent with Zamanian et al. [40]. They reported a reduction of water vapor penetration when TiO_2_ nanoparticles are embedded into neat polyvinyl alcohol films. In addition, when a small amount of Closite 20A (3%) was incorporated, the water contact angles of PE-C and PE-CT significantly increased. This implies a decrease in the degree of wetting for PE-C and PE-CT samples due to the exfoliation of layered silicates, making the surface became less hydrophilic. These findings are align with those of Akin and Tihminlioglu [41], who reported that, the water contact angles of polyhydroxybutyrate (PHB) nanocomposites significantly increased with the addition of 1% and 2% of Closite 10A, reaching its highest value of 81.62°, in comparison to pure PHB sample. The contact angle measurements exhibited a consistent trend that aligned well with the water vapor permeability measurements, providing support for the notion of the influence of dispersion level on surface hydrophilicity reduction.

As noted by Kawasuni [42], the presence of nonpolar groups in the polyolefin backbone and the inherent polarity of clay silicate layers, even when modified by nonpolar long alkyl groups, result in incompatibility with polyolefin. Hence, it is crucial to investigate the diverse parameters and variables entailed in the development of polyolefin nanocomposites. In this specific instance, as illustrated in Fig. 2, the optimization of the process enabled the creation of an exfoliated nanocomposite film.

It is widely recognized that the respiration rate of commodities increases during postharvest storage. The deceleration of the respiration rate can effectively extend storage life [43]. The creation of a low-O_2_ and high-CO_2_ atmosphere in PE-CT and PE-C could provide an optimal composition for tomato fruit storage, inducing significant changes in enzymatic activities related to respiratory metabolism and potentially inhibiting oxidative phosphorylation [44]. The results suggest that the modified atmosphere mechanism in PE-CT and PE-C plays a crucial role in extending the storage life of tomato fruit compared to PE-T and PE. It also confirms the results obtained from the permeability analysis of the films mentioned in the permeability analysis section. These findings align with those of Li et al. [45], who reported that nanocomposite packaging films with modified barrier properties against O_2_ and CO_2_ effectively preserved the overall quality of fresh strawberry fruits.

The plausible mechanism through which TiO_2_ nanoparticles may have curtailed ethylene production entails the catalytic decomposition or oxidation of ethylene into water and CO_2_. The findings by Antunes et al. [46] postulate that the initiation of autocatalytic ethylene production via free radicals could potentially expedite postharvest ripening and senescence in fruits. Consequently, PE-T and PE-CT configurations demonstrated promise in abating ethylene generation due to the catalytic potential of TiO_2_ nanoparticles in ethylene oxidation. Furthermore, the reduction of ethylene production in nanocomposite films containing clay nanoparticles can be attributed to the decreased permeability of these films and the higher concentration of CO_2_ inside the packages. A high concentration of CO_2_ inhibits ethylene biosynthesis, as well as reduces the abundance of ATP and prevents ACC synthase and ACC oxidase activity [47]. These outcomes align with the earlier investigations on kiwifruit conducted by Hu et al. [19] while in previous studies, the effect of using nanoparticles on ethylene production of tomato fruit during postharvest storage has not been reported [22, 23].

An increase in CO_2_ concentration in the headspace of packed tomato fruit can potentially lead to reduced fruit respiration over time, subsequently extending the shelf life of packed fruits [48]. Weight loss is considered a significant determinant in the post-harvest life and fresh produce quality. An acceptable weight loss during the storage period falls within the range of 6–7% [49]. In the present study, a substantial difference in weight loss was observed between tomatoes packed with PE and nanocomposite films compared to the aforementioned acceptable range. Although the creation of a modified atmosphere due to reduced fruit respiration has mitigated weight loss during the storage period, the addition of nanoparticles such as clay and TiO_2_ to the polymer matrix of polyethylene films (PE-T, PE-C, and particularly PE-CT) appears to have significantly reduced gas permeability in comparison to PE film. The findings of this study are in line with previous reports on the utilization of nanocomposite films for kiwifruit and pear [19, 21].

The remarkable reduction in fruit firmness could be attributed to the decrease in fruit tissue water content and the activation of cell wall softening enzymes, including polygalacturonase [19, 50]. In this study, the rate of fruit softening was influenced by the modified atmosphere and the nanoparticles present in the nanocomposite film. The findings of this research align with reports on the efficacy of various polyethylene films in preserving the quality and tissue firmness of pear fruits [51] and, the packaging of pear and strawberry fruits using nanocomposite films [19, 21].

As depicted in Table 3, the highest TSS changes were observed in the control treatment (unpacked) even on day 35, compared to the changes in packaged fruit treatments. The least variation in TSS was attained with the application of the nanocomposite films compared to the PE film. In packed fruits, these changes in PE-CT and PE treatments were 37% and 43%, respectively, compared to the value at the beginning of the experiment.

The utilization of packaging and the creation of a modified atmosphere revealed statistically significant differences in TSS values among treated fruits. TSS quantity was considered as a crucial indicator of tomato maturation. The variations in TSS during the fruit ripening process are associated with starch hydrolysis and its conversion into simple sugars [52]. The increase in pH levels can be attributed to the consumption of organic acids present in the fruit, which is a consequence of the respiratory process [53].

Typically, over time during the storage period, sugars and organic acids decrease due to metabolic activities such as respiration, leading to changes in TSS, TA, and pH levels [54]. The reduction in variations of the mentioned attributes in fruit packed with PE and nanocomposite films is linked to increased CO_2_ levels and reduced respiration intensity associated with the increased impermeability of packaging films and the photocatalytic property of TiO_2_ nanoparticles [55]. It seems that the addition of nanoparticles, such as clay and TiO_2_, along with the reduced permeability of packaging films and the photocatalytic property of TiO_2_ nanoparticles, has significantly decreased the ethylene production in the internal atmosphere of packed tomato fruit, consequently leading to remarkable attenuation of treated fruit softening. This suggests the likelihood of more controlled fruit respiration in the PE-CT film compared to other films.

The outcomes achieved through the application of a modified atmosphere combined with 1-MCP in tomato fruits corroborate the present study's findings, indicating the reduction of physiological processes involved in fruit ripening and alterations in TSS, TA, and pH levels [48].

The results demonstrated that the employment of clay and TiO_2_ nanoparticles effectively contributed to membrane stability preservation and inhibition of MDA accumulation. A portion of membrane destabilization during the ripening process in fruit and vegetable products at the postharvest stage is attributed to lipid peroxidation of fatty acids. The extent of lipid peroxidation can be assessed by the level of MDA production [56]. Two suitable indicators for evaluating damages resulting from oxidative reactions due to stress and aging processes are electrolyte leakage and the amount of MDA produced in fresh products [57]. The intensity of damage and cellular membrane disruption is proportional to the increase in electrolyte leakage [13]. Lipid peroxidation arises from the accumulation of reactive oxygen species (ROS), leading to membrane damage, increased electrolyte leakage, and elevated MDA levels [58].

The present research outcomes affirmed the efficacy of optimized films enriched with clay and TiO_2_ nanoparticles in preventing the escalation of MDA and electrolyte leakage in tomato fruits. The applicability of nanomaterials in maintaining membrane stability and reducing MDA production in kiwifruit packed with nanocomposite films under low-temperature storage conditions has also been reported [19].

The progress of the senescence process in fruits is accompanied by an increase in the production of ROSs. To regulate oxidative stress, accumulated H_2_O_2_ resulting from the aging process needs to be converted into non-toxic molecules through antioxidant enzymes such as CAT and APX [59]. The reduction in CAT and APX enzyme activities in unpacked fruits could be attributed to cold injury during low-temperature storage of tomato fruits. This observation aligns with the prior discovery which documented reduced antioxidant activities in pepper and peach fruits under cold stress conditions [20, 60]. The application of modified atmosphere packaging can better preserve the activity of antioxidant enzymes and subsequently reduce cold injury by maintaining the moisture content inside the package around the produces, slowing down the ripening process, and preventing plasma membrane damage [61]. In the current study, the simultaneous addition of clay and TiO_2_ nanoparticles led to further protection of antioxidant enzyme activities compared to individual applications of these nanoparticles. The ability of clay nanoparticles to optimize film permeability is one contributing factor. Another factor is the role of TiO_2_ nanoparticles as photo-catalysts, which reduces ethylene production in the internal atmosphere of the film. This, in turn, decreases the activity of spoilage microorganisms, collectively contributing to the preservation of antioxidant enzyme activities until the end of the experimental period. The findings of this research are in line with the results obtained from the application of nanocomposite films containing clay and TiO_2_ nanoparticles, demonstrating a reduction in ethylene production in treated fruits and the preservation of antioxidant enzyme activities in strawberry, pear, and peach fruits [20, 21, 45].

In this study, the highest inhibition of fungal population growth was observed in the PE-CT nanocomposite film, which can be attributed to the positive interaction of TiO_2_ nanoparticles due to their photocatalytic properties and the localized antimicrobial effects of clay nanoparticles upon direct contact with the packed fruits. Excited electrons on the surface of TiO_2_ nanoparticles, induced by ultraviolet light, can generate hydroxyl radicals and ROS, which possess oxidizing capabilities to eliminate spoilage microorganisms [10].

## Conclusion

The incorporation of clay and TiO_2_ nanoparticles into LDPE films has proven to be a promising approach for enhancing the mechanical strength, gas barrier properties, and quality attributes of tomato fruits. The synergistic interaction between these nanoparticles has led to remarkable improvements in mechanical properties, reduced gas permeability, enhanced membrane stability, and reduced oxidative stress in packed tomatoes. The preservation of antioxidant enzyme activities, inhibition of fungal decay, and mitigation of ethylene production further validate the effectiveness of these nanocomposite films in extending the storage life of tomato fruits. The findings from this study hold potential implications for the development of advanced packaging materials that can significantly improve the postharvest preservation of fresh produce.

## Data Availability

The data that support the findings of this study are available from the corresponding author upon reasonable request.
